# Orchestration of intestinal homeostasis and tolerance by group 3 innate lymphoid cells

**DOI:** 10.1007/s00281-018-0687-8

**Published:** 2018-05-08

**Authors:** Hugo A. Penny, Suzanne H. Hodge, Matthew R. Hepworth

**Affiliations:** Manchester Collaborative Centre for Inflammation Research (MCCIR), Division of Infection, Immunity and Respiratory Medicine, School of Biological Sciences, Faculty of Biology, Medicine and Health, Manchester Academic Health Science Centre, University of Manchester, Manchester, M13 9PL UK

**Keywords:** Innate lymphoid cells, ILC, Mucosal tolerance, Commensal bacteria, Inflammation, Inflammatory bowel disease, Dietary antigens

## Abstract

The gastrointestinal tract is the primary site of exposure to a multitude of microbial, environmental, and dietary challenges. As a result, immune responses in the intestine need to be tightly regulated in order to prevent inappropriate inflammatory responses to exogenous stimuli. Intestinal homeostasis and tolerance are mediated through a multitude of immune mechanisms that act to reinforce barrier integrity, maintain the segregation and balance of commensal microbes, and ensure tissue health and regeneration. Here, we discuss the role of group 3 innate lymphoid cells (ILC3) as key regulators of intestinal health and highlight how increasing evidence implicates dysregulation of this innate immune cell population in the onset or progression of a broad range of clinically relevant pathologies. Finally, we discuss how the next generation of immunotherapeutics may be utilized to target ILC3 in disease and restore gastrointestinal tolerance and tissue health.

## Introduction

Mammalian mucosal barrier sites, such as the gastrointestinal tract and lung, are primary sites of exposure to a wide range of exogenous organisms and environmentally derived antigens and ligands. In particular, the gastrointestinal tract is constantly exposed to a complex range of micro- and macroorganisms with the potential to establish either stable mutualistic relationships within the host or, conversely, to drive potentially life-threatening disease. A prime example of this is the trillions of bacteria that compete for space and nutrients within the gastrointestinal tract. While the majority of these bacteria enjoy a harmless or even beneficial, symbiotic relationship within the host and form a microbial community known as the commensal microbiota, some species have inherent pathogenicity and the capacity to cause serious illness [1]. Furthermore, the gastrointestinal tract is also increasingly understood to play host to a fungal “mycobiome” [2], a significant number of viruses and bacteriophages [3], and in a significant proportion of humans worldwide—gastrointestinal helminths [4]. In addition to the abundance of foreign organisms co-existing within the gastrointestinal tract, the gut is also the major site of exposure to a broad range of dietary proteins, lipids, carbohydrates, and phytochemicals that have the potential to elicit immune responses [5, 6]. Thus, for the host to maintain tissue health and thrive, it is vital that the intestinal immune system is tightly regulated. This requires maintaining the ability to generate protective immunity against pathogens while simultaneously establishing a state of tolerance towards the diet and beneficial commensal organisms.

Gastrointestinal homeostasis and immune tolerance are mediated through a wide range of complementary mechanisms that together prevent immune responses to innocuous stimuli and subsequently suppress inflammation and tissue damage. The maintenance of tissue homeostasis and tolerance at mucosal barrier sites is orchestrated chiefly by cells of the immune system, which reinforce the integrity of the epithelial barrier and regulate other lymphocytes and myeloid cells within the tissue microenvironment through dynamic cross talk. Although many tolerogenic functions have been attributed to both the adaptive and innate immune system, recent advances have begun to highlight the importance of innate lymphoid cell (ILC) family members in maintaining healthy interactions between the host and the wide range of challenges faced in the gastrointestinal tract.

ILC are a family of tissue-resident, transcriptionally poised effector lymphocytes that respond rapidly to tissue-damage-associated danger signals and microbially induced signals by potently secreting cytokines which promote pathogen killing, wound healing, and barrier function [7–11]. In contrast to adaptive lymphocytes, ILC lack rearranged antigen-specific receptors and differ from many innate immune cells as they largely lack the machinery to directly sense microbial patterns (i.e. Toll-like receptors (TLR)). ILC canonically comprise several fully polarized subsets that mirror CD4^+^ T helper cell phenotypes, develop from a common progenitor and are defined by their lack of lineage markers that are expressed by T, B, and myeloid cell populations as well as their common expression of the interleukin (IL)-7Rα chain. ILC are further subdivided into distinct groups by transcription factor expression and cytokine production and include T-bet^+^ IFN-γ producing group 1 (ILC1), GATA-3^hi^ IL-5 and IL-13 producing group 2 (ILC2), and RORγt^+^ IL-17 and IL-22 producing group 3 (ILC3) (ILC nomenclature and development have been reviewed extensively in references 7–11). In addition, a distinct regulatory ILC subset (ILCreg) has recently been described which appears to have important functions in preventing inflammation in the gastrointestinal tract [12]. Although other ILC subsets likely play key roles in tissue repair, ILC3 are emerging as central to the maintenance of gastrointestinal homeostasis and tolerance. In this review, we will discuss the increasing number of mechanisms through which ILC3 are thought to mediate host interactions with microbes, diet, and the external environment and discuss evidence implicating dysregulation of ILC3 biology in human inflammatory disorders that directly result from loss of mucosal tolerance.

## ILC3 in tissue homeostasis and intestinal tolerance

### ILC3 subsets in the gastrointestinal tract and associated lymphoid tissue

ILC3 are characterized by expression of the transcription factor retinoic acid related orphan receptor gamma isoform t (RORγt) and production of the cytokines IL-17A, IL-17F, IL-22 and granulocyte-macrophage colony-stimulating factor (GM-CSF) [8–10, 13, 14]. In mice, ILC3 consist of at least two distinct subsets. Lymphoid tissue inducer (LTi)-like ILC3 are distinguished by their expression of CCR6 and c-kit, heterogeneous expression of CD4, and localization within lymphoid tissues. In contrast, natural cytotoxicity receptor-expressing (NCR^+^) ILC3 lack LTi-associated markers (i.e. CCR6) and are instead distinguished by their expression of NKp46 and CD49a and co-expression of the transcription factor T-bet and reside largely within the small intestinal lamina propria [8–10, 13, 14]. Similarly, in humans, multiple ILC3 subsets have been defined through phenotypic and single-cell transcriptomic studies [15, 16], although whether these populations are fully analogous to those found in the murine gut remains unclear. Nonetheless, in both mice and humans, these subsets localize to different tissue niches and are developmentally, transcriptionally, and functionally different [10, 14]. In particular, emerging evidence suggests ILC3 subsets have the capacity to respond to differential cues within tissues and have differential roles—with NCR^+^ ILC3 implicated in inflammatory responses through their T-bet dependent ability to produce IFN-γ, while LTi-like ILC3 appear to be enriched for homeostatic functions, and endowed with the ability to regulate the adaptive immune system (reviewed in detail below) [10, 14, 17, 18]. Thus, further understanding of the specific roles mediated by ILC3 subsets may be critical for the treatment of multiple intestinal inflammatory disorders and for the development of new clinical strategies for therapeutic intervention.

**Fig. 1:**
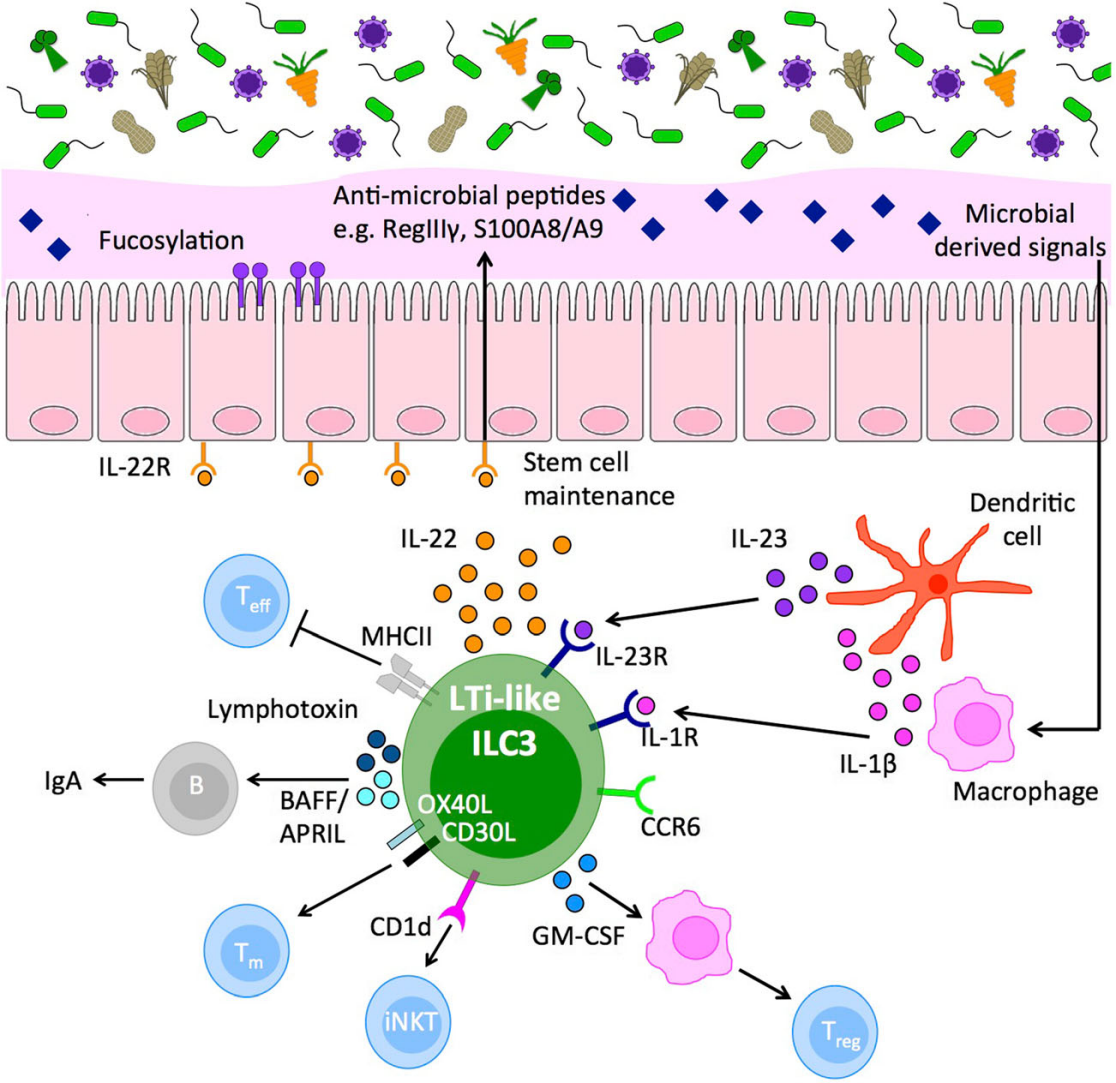
Group 3 innate lymphoid cells are central orchestrators of intestinal immune tolerance. The intestinal tract is host to a wide range of exogenous antigenic stimuli derived from microbial and dietary sources. Group 3 innate lymphoid cells (ILC3) are present constitutively in the intestine and have central roles in maintaining tolerance and tissue homeostasis. In particular, one subset of ILC3 (LTi-like ILC3) possess multiple mechanisms to regulate immune responses and reinforce intestinal barrier function in the intestinal tissue and associated lymphoid structures. Intestinal ILC3 are a dominant source of interleukin (IL)-22 at steady state, produced in response to microbially driven cytokine cues provided by tissue-resident myeloid cells. IL-22 acts to reinforce epithelial barrier tight junctions and induce antimicrobial peptides, mucus production, and fucosylation of the epithelial cells to maintain segregation of commensal microbes. In addition, ILC3 act either indirectly (via cytokine effects on intermediary cells) or directly (via cell–cell contact) to control the quality and magnitude of the adaptive immune response. Together, intestinal ILC3 represent an important cellular regulator of intestinal tissue health

ILC3 subsets are constitutively present in both murine and human intestine although their relative distribution within the tissue and along the length of the gastrointestinal tract appears to differ. For example, LTi-like ILC3 are largely found within secondary and tertiary lymphoid structures including the intestinal draining (mesenteric) lymph node, Peyer’s patches, cryptopatches, and isolated lymphoid follicles (ILFs), where they localize within microniches in close proximity to adaptive immune cells, including CD4^+^ T cells and B cells (Fig. 1) [19]. Although the ontological relationship of adult LTi-like ILC3 with de facto fetal LTi cells—which orchestrate lymphoid tissue formation during embryogenesis—remains unclear, their localization in lymphoid structures suggests this ILC3 subset preferentially resides within well-organized and defined microenvironments in both the small and large intestines. LTi-like ILC3 express a specific range of chemokine receptors including CCR6, CXCR5, and CCR7 [20–22], although the relative role and full extent to which these receptors contribute to LTi-like ILC3 localization have not been fully elucidated. However, expression of CCR7 is required for the migration of LTi-like ILC3 between the intestine and mesenteric lymph node (mLN) [21]. Interestingly, another homing receptor, *Gpr183* (EBI2), was recently shown to be essential for LTi-like ILC3 residence within cryptopatches and ILFs in the gut [23]. In contrast, murine NCR^+^ ILC3 predominantly reside within the murine small intestine and appear to be largely excluded from lymphoid tissues, particularly the intestinal draining lymph node. In this regard, NCR^+^ ILC3 preferentially express the chemokine receptor CXCR6 [24], which acts to retain cells within the intestinal lamina propria.

### Homeostatic cytokine production by ILC3 subsets

ILC3 are a potent and dominant source of IL-22 under homeostatic conditions, and tonic production of this cytokine is increasingly appreciated to be a critical regulator of intestinal health and immune homeostasis [13, 25–29]. Both NCR^+^ and LTi-like ILC3 have the capacity to produce IL-22 in response to myeloid cell-derived activating signals, including IL-1β and IL-23 [30]. Multiple studies have historically attributed roles to IL-22 derived from both ILC3 subsets, particularly in mediating protective immunity to the enteric bacterial pathogen *Citrobacter rodentium* [25, 28, 31, 32]. However, recent transgenic approaches to dissect the contribution of NCR^+^ versus LTi-like ILC3-derived IL-22 in response to this pathogen have suggested NCR^+^ ILC3-derived cytokine is likely not required to control bacterial load [33, 34], in line with the dominance of LTi-like ILC3 in the colon—the site of infection—and previous reports suggesting LTi-like ILC3-derived IL-22 is critical in this model [28]. Despite these advances, the precise relative contribution of IL-22 derived from the two ILC3 subsets to intestinal tissue homeostasis remains incompletely understood, and it is notable that many seminal studies have utilized mice lacking the adaptive immune system (e.g. *Rag1*^−/−^) to dissect the contribution of ILC3-derived cytokines. Thus, these studies highlight the need for new experimental approaches that allow for specific and selective depletion of ILC3 subsets in the presence of adaptive immunity. Nonetheless, IL-22 from ILC3 clearly plays critical roles in preventing inappropriate immune responses to microbial and environmental antigens. The protective effect of this axis is most notably described in the maintenance of healthy host interactions with the commensal microbiome. Loss of ILC3-derived IL-22 results in translocation of commensal bacteria to peripheral organs, such as the spleen and liver, and outgrowth of bacterial species which can successfully establish residence close to the epithelium or within lymphoid tissues [25, 29, 35–37]—indicating a critical role for this cytokine in maintaining intestinal barrier function (Fig. 1).

The intestinal epithelium and mucus layer are the primary lines of defense in the gut and act not only as a physical barrier but also as an active regulator of commensal bacterial colonization and species diversity. IL-22 acts directly via its receptor, which is expressed solely on non-hematopoietic cells—including intestinal epithelial cells (IECs), by phosphorylating the transcription factor STAT3 and modulating gene expression [13]. IL-22-producing ILC3 resident within cryptopatches act to reinforce epithelial barrier homeostasis in part through maintenance of crypt stem cells, which give rise to IECs and other specialized epithelial and enteroendocrine cell subsets [38–40]. In addition, IL-22 promotes differentiation of mucus-producing goblet cells [41, 42], which are critical in forming the viscous mucus layer that acts to maintain physical segregation of the majority of the commensal microbiota from the underlying epithelia and immune cells present within the lamina propria. Moreover, in response to IL-22, IECs and specialized Paneth cells are stimulated to produce high levels of antimicrobial peptides including S100A8/A9, which compete with potentially invasive microbial strains for vital nutrients and essential metals, and RegIIIγ which acts to maintain bacterial segregation by diffusing within the inner mucus layer to directly kill bacterial species with invasive potential [13, 43–46]. In addition, IL-22 induces fucosylation of epithelial cell-associated carbohydrates—an adaptation that promotes the growth of mutualistic commensal species that have adapted to utilize fucose as an energy source and subsequently outcompete pathogenic bacteria that lack this ability [47–49]. ILC3-derived cytokines, including IL-17A and IL-22, are also thought to have critical roles in responses against fungal pathogens, such as *Candida albicans* [50, 51]. However, whether constitutive production of IL-22 at homeostasis plays similar roles in controlling the constituents of the fungal mycobiome, as it does with the microbiome, remains unclear [2]. Nonetheless, commensal fungal colonization of the intestine is thought to induce many of the same immune pathways as the bacterial microbiota and protects from intestinal inflammation [2, 52–54], providing a strong rationale for investigating the role of ILC3-derived cytokines in regulation of fungal communities and tolerance to the commensal mycobiota further.

### Beyond the microbiota: cross talk between ILC3 and diet in intestinal tolerance

Although much is known about the role of ILC3-derived IL-22 in maintaining intestinal homeostasis in the presence of the microbiota, relatively little is known about how this axis regulates responses against dietary antigens. ILC3 are acutely susceptible to modulation by dietary-derived vitamins and phytochemicals. The vitamin A metabolite retinoic acid is critical for maintenance of ILC3 subsets in the adult intestine, while maternal retinoids are also critical for bona fide LTi cell maturation and lymphoid tissue formation in utero [55–57]. Similarly, vitamin D also acts to modulate ILC3 function and has been demonstrated to attenuate IL-23R signaling and suppress cytokine production following activation [58]. Moreover, aryl hydrocarbon receptor (Ahr) ligands—present in cruciferous vegetables and produced by the microbiota—are required for the development and seeding of the intestine by both ILC3 populations and optimal production of IL-22 [32, 59–61]. The requirement for Ahr signaling is particularly pronounced in the early colonization of the gastrointestinal tract by NCR^+^ ILC3 following birth, a process in-part dependent upon transfer of maternal antibodies bound to Ahr ligands [62, 63]. Thus, ILC3 sensing of dietary components indicates these cells may be poised to modulate host tolerance of food-derived antigens. In line with this, a cross talk between bacterial colonization, ILC3, and tolerance to dietary antigens has been described [64]. In this study, colonization by a *Clostridia* spp. containing microbiota resulted in activation of ILC3 and production of IL-22, which subsequently reduced uptake of dietary antigen by epithelial cells and reduced sensitization and development of food allergy [64]. In addition to a broad range of food allergies, breakdown of intestinal tolerance has been associated with inflammation driven by dietary gluten, as seen in celiac disease patients. Inflammatory ILC producing IFN-γ and TNF-α have been described in biopsies taken from celiac disease patients [65]; however, it is currently unclear whether ILC3-derived cytokines—particularly IL-22—maintain tolerance to dietary gluten under homeostatic conditions. In this regard, ILC3 have been attributed a critical role in establishing oral tolerance to dietary antigens through the production of the cytokine GM-CSF, which subsequently supports the activity of tolerogenic intestinal mononuclear phagocyte populations with the ability to promote differentiation of regulatory T cells (Treg) [66]. Thus, it is increasingly appreciated that ILC3 are key mediators of intestinal tolerance, in particular via production of cytokines. However, emerging evidence suggests ILC3 mediate their tolerogenic effects through multiple other pathways, while an increasing understanding of the role of these pathways in broader intestinal tolerance to fungal and dietary-derived antigens could provide opportunities for novel therapeutic interventions.

### ILC3 regulation of adaptive immunity and mucosal tolerance

While ILC3 mediate many of their regulatory effects through effector cytokines, an increasing body of evidence suggests ILC3 also directly modulate and orchestrate the adaptive immune response in order to maintain intestinal health [10, 67, 68]. In particular, an increasing number of studies have described roles for LTi-like ILC3 in directly interacting with cells of the adaptive immune system and, unlike NCR^+^ ILC3, LTi-like ILC3 are endowed with multiple mechanisms to modulate both T and B cells. For example, LTi-like ILC3 in the mLN and colon express constitutively high levels of major histocompatibility complex class II (MHCII) and present antigen to CD4^+^ T cells [69–71]. In contrast to other canonical antigen-presenting cells, T cell receptor (TCR) engagement by MHCII^+^ LTi-like ILC3 results in T cell death—due in part to the absence of co-stimulatory molecules such as CD80 and CD86 and the ability of LTi-like ILC3 to outcompete T cells for local IL-2, needed for T cell proliferation [69, 70]. Antigen presentation by LTi-like ILC3 was found to be critical for tolerance to commensal bacteria as mice with an ILC3-intrinsic deletion of MHCII developed T cell-driven colitis dependent upon the intestinal microbiota [69, 70]. Conversely, LTi-like ILC3 express non-classical ligands for T cell interaction including OX40L and CD30L, which act to support memory T cell survival [72–74]. LTi-like ILC3 also have the ability to modulate innate-like T cell populations, such as invariant NKT cells, through the presentation of lipid antigens on the antigen-presenting molecule CD1d [75], further indicating these cells act as critical regulators of the adaptive immune system at homeostasis. Together, these studies suggest a more nuanced model of LTi-like ILC3 regulation of T cell responses whereby these cells may act as a checkpoint in lymphoid tissues to prevent aberrant inflammatory responses while maintaining long-term memory populations required for optimal immunity. Further studies and novel experimental approaches are needed to clarify whether antigen presentation and interactions between LTi-like ILC3 and T cells occur solely within the draining lymph nodes, within tissue-associated tertiary lymphoid structures (e.g. Peyer’s patches, cryptopatches, ILF), or both.

In addition to their ability to present antigen and provide auxiliary signals to T cell populations, an increasing body of evidence also suggests LTi-like ILC3 are critical regulators of steady-state B cell responses. B cell responses are central to intestinal tolerance due to their ability to produce high quantities of immunoglobulin A (IgA). Secretion of IgA across the intestinal barrier enhances the physical segregation of commensal bacteria, controls the balance of commensal species that establish residence in the intestinal microenvironment and neutralizes potentially harmful bacterial toxins and dietary products [76]. The majority of IgA produced under steady-state circumstances is produced by innate-like B cells and does not require interactions between B cells and specialized follicular helper T cells (TfH) [77, 78]. LTi-like ILC3 are critical in the production of innate, tolerogenic IgA in the intestine through a range of indirect and direct mechanisms. LTi-like ILC3 are a potent source of surface-bound and secreted lymphotoxin, which is required for the formation of tertiary lymphoid tissues such as Peyer’s patches, cryptopatches, and ILFs where the induction of IgA-secreting innate B cell populations takes place [79–81]. Additionally, LTi-like ILC3 provide a source of survival signals and secreted factors, including BAFF/APRIL and delta-like ligand 1 (*Dll1*), which promote B cell class switching and antibody production [82]. Moreover, in humans, ILC3 provision of BAFF and CD40L-dependent interactions with B cells favors the development of an IL-10-producing regulatory B cell population [83]. Taken together, these studies highlight LTi-like ILC3 as key orchestrators of adaptive immune responses in lymphoid tissues and implicate regulation of T and B cell responses by ILC3 in the maintenance of immune homeostasis and intestinal tolerance (Fig. 1).

## Loss of tolerance: dysregulated ILC3 responses in human disease

### Inflammatory bowel disease

**Fig. 2:**
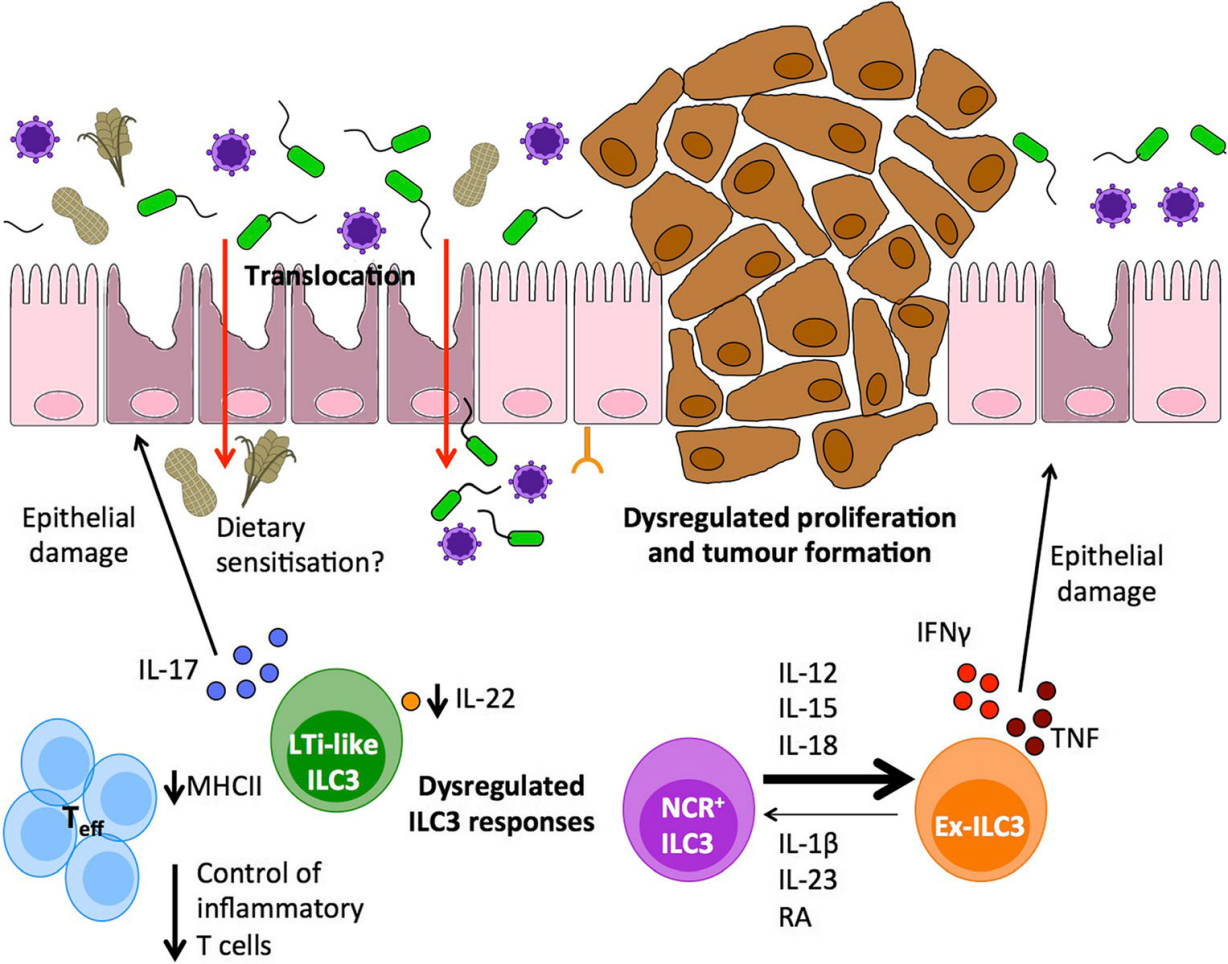
Dysregulated ILC3 responses precipitate the onset and progression of gastrointestinal disease. A broad range of intestinal pathologies are inherently associated with a loss of intestinal tolerance and dysregulated inflammatory immune responses. Dysregulation of intestinal ILC3 numbers and functions has been implicated in the pathogenesis and progression in multiple clinically relevant diseases including inflammatory bowel disease (IBD), HIV infection, graft-versus-host disease (GVHD), and colorectal cancer (CRC). These pathologies have been associated with a loss of ILC3-intrinsic homeostatic functions including antigen-presenting function and IL-22 production and subsequent failure to maintain the intestinal barrier—resulting in antigen translocation and sensitization of the underlying immune system and aberrant inflammatory T cell responses. Furthermore, dysregulation of IL-22 signaling leads to epithelial hyperplasia that can progress to colorectal cancer in the presence of chronic inflammation. Thus, therapeutic strategies to restore homeostatic ILC3 functions may prove efficacious in intestinal inflammatory disorders. In addition, NCR-expressing ILC3 (NCR^+^ ILC3) exhibit a degree of functional and transcriptional plasticity and in the presence of an inflammatory cytokine milieu lose RORγt expression to become ILC1-like “ex-ILC3,” a phenotype associated with production of pro-inflammatory cytokines such as IFN-γ and which has been implicated in driving intestinal pathology and tissue damage in IBD. Thus, therapeutic interventions to suppress inflammatory ILC3 may offer a novel strategy to promote resolution of disease in multiple intestinal disorders

While ILC3 appear to play critical roles in maintaining intestinal tolerance and tissue homeostasis, dysregulation of ILC3 function resulting in loss of beneficial pathways and/or inflammatory ILC3 responses have been associated with a broad range of human chronic inflammatory diseases (Fig. 2). This paradigm is typified in inflammatory bowel disease (IBD), a chronic inflammatory disorder of the GI tract that encompasses two main forms, Crohn’s disease (CD) and ulcerative colitis (UC), and which results in inflammation and significant tissue damage [1, 84–86]. The etiology of IBD is complex and incompletely understood, but available evidence suggests that IBD is caused by genetic and environmental influences that result in an inappropriate immune response against intestinal commensal bacteria in genetically susceptible individuals, and that disease involves dysregulation of both innate and adaptive immunity [1, 84–86]. Genome-wide association studies (GWAS) have identified networks of genes involved in mediating interactions with the host microbiota—including multiple ILC3-associated genes—as risk alleles for IBD [85, 87, 88]. For example, IBD has been associated with polymorphisms in genes heavily associated with ILC3 transcription and phenotype (*Rorc*, *Nfil3*), effector function and signaling (*Il22*, *Il23r*, *Il1r1*, *Il2ra*, *Il15ra*, *Il2*, *Stat5a/b*) and migration (*Cxcr5*, *Gpr183*, *Ccr6*) [85, 87, 88]. Together, these associations suggest multiple lesions in ILC3-associated genes may act to predispose to the development and/or progression of IBD and provoke the hypothesis that dysregulated ILC3 responses may play key roles in the pathogenesis of IBD.

This hypothesis is further supported by a range of both human and mouse experimental studies, which together indicate IBD is associated with both a loss of beneficial ILC3 functions that usually promote immune homeostasis and tolerance, as well as dysregulation of ILC3 that results in an inflammatory phenotype which may act to exacerbate disease. For example, patients with CD have been shown to have lower frequencies of lamina propria IL-22-producing NKp44^+^ ILC3s [89, 90], suggesting this protective pathway may be disrupted. Similarly, pediatric CD patients exhibited reduced expression of MHCII (HLA-DR) on ILC3 taken from intestinal biopsies suggesting regulatory antigen-presenting function may be disrupted in some cohorts of IBD patients [69]. Conversely, ILC3 have been suggested to play disease-driving roles in colitis through the production of inflammatory cytokines. The number and activity of IL-17 and IFN-γ producing ILC3s have been reported to be increased in the inflamed ileum and colon of patients with CD and in mouse models of bacteria-driven colitis [91, 92]. As discussed earlier, this could be in part due to differential functions of NCR^+^ and LTi-like ILC3 subsets. Indeed, unlike LTi-like ILC3, NCR^+^ ILC3 co-express the transcription factor T-bet and have the capacity to produce IFN-γ and TNF-α [17, 18, 93]. Moreover, NCR^+^ ILC3 have the potential to acquire an inflammatory phenotype in the context of intestinal inflammation, associated with increased T-bet expression and loss of RORγt expression [17, 94]. Interconversion of NCR^+^ ILC3 to IFN-γ producing “ex-ILC3s” is determined by the local cytokine milieu and has been demonstrated to drive colitis pathology in mice and associated with disease in CD patients [18, 89, 95–97]. Importantly, this phenotypic plasticity is reversible upon resolution of inflammation and driven by homeostatic cytokine signals, suggesting blockade of inflammatory ILC polarizing cytokines may have therapeutic potential and favor reversion to a homeostatic phenotype.

Given the emerging prominence of ILC3 as key innate immune cells in the pathogenesis of colitis, there is an increasing interest in the possibility of targeting these cells for the treatment of IBD. This is particularly feasible due to the current pipeline of novel therapeutics being developed to target Th17 cells—which are key inflammatory cells in IBD patients—and which share transcription factors and common up- and downstream pathways with ILC3 [98, 99]. However, any approaches broadly targeting ILC3 function have the potential to disrupt both beneficial LTi-like ILC3-mediated tolerogenic effects as well as inflammatory ILC3 responses, which could result in long-term consequences and unforeseen side effects. One possible approach would be to utilize small-molecule antagonists specific for RORγt, which have been demonstrated to have efficacy in human Th17 cells [100, 101]. In this regard, the effect of small-molecule RORγt inhibitors on ILC3 responses has recently been investigated. Surprisingly, while these antagonists were efficient in reducing IL-17A production by Th17 cells in intestinal biopsies from IBD patients, they had no effect on ILC3 IL-22 production [102]. In line with this finding, inducible deletion of RORγt in ILC3 using transgenic mice failed to disrupt ILC3 survival, MHCII-expression or IL-22 production, suggesting that mature ILC3 do not require this transcription factor for their persistence in the periphery, whereas Th17 cells are rapidly lost in the absence of this transcription factor [102]. Moreover, these findings imply that, despite shared transcriptional networks and effector functions, strategies to target inflammatory Th17 in disease may not necessarily suppress beneficial, homeostatic ILC3 function.

Additional approaches utilizing monoclonal antibody-based therapies have the potential to revolutionize treatment for IBD. Antibodies targeting IL-23 (e.g. *Ustekinumab*, *Tildrakizumab*, *Guselkumab*), IL-17A (e.g. *Secukinumab*, *Ixekizumab*) and IL-17R (e.g. *Brodalumab*) have been developed for use in the clinic due to their effects on inflammatory Th17 cells [98]. However, it is nonetheless important to consider the potential effects of these treatments on ILC3. In particular, it is notable that current target cytokines also have important beneficial roles in the intestine either by promoting barrier homeostasis (e.g. IL-23–IL-22 axis) or through regulation of commensal microbes (e.g. IL-17A–IL-17R axis), and the effects of these cytokines are often context dependent. In line with this, targeting IL-17A with secukinumab was found to be deleterious for Crohn’s patients [103], potentially due to the inhibition of protective roles for IL-17A in the intestine. Indeed, IL-17A regulates the epithelial barrier function and acts to control bacterial and fungal populations with the potential to drive intestinal inflammation [51, 104]. In contrast, administration of ustekinumab which neutralizes IL-12p40, a subunit of both IL-12 and IL-23, successfully reduces inflammation in a proportion of CD patients [105]. However, the anti-inflammatory properties of this monoclonal antibody may be attributed to the concurrent inhibition of IL-12-driven, IFN-γ mediated inflammatory pathways including plastic populations of inflammatory Th1 and ILC1/“ex-ILC3” [95, 106]. Thus, dysregulated ILC3 responses are increasingly being associated with IBD pathogenesis and may be amenable to therapeutic intervention, but the potential impact on beneficial and regulatory ILC3 responses needs to be considered in the context of potential long-term implication. Studies aimed at elucidating the full extent to which the biology of NCR^+^ ILC3 and LTi-like ILC3 differs may allow for the identification of novel therapeutic avenues aimed at maintaining homeostatic functions and suppressing inflammatory pathways.

### Colorectal cancer

IBD patients are at an increased risk of developing colorectal cancer (CRC), an association thought to be a consequence of chronic intestinal inflammation. ILC3 have been reported to infiltrate tumors in multiple tissues and have been associated with the development, progression and control of tumorigenesis in multiple human cancers, most notably colorectal cancer, a finding that is supported by a growing body of experimental evidence from mouse models of colon cancer (reviewed in detail in [107, 108]). The activation status of ILC3 and their cytokine production appear to be central to their impact on colorectal cancer. Elevated expression of IL-23 has been observed in human colon tumors in comparison to healthy tissue and has been linked to an adverse prognosis and more aggressive disease [109], while mice deficient for IL-23 are resistant to tumor formation [109–111]. Conversely, transgenic overexpression of IL-23 in wild-type mice results in adenoma formation in the absence of exogenous carcinogens, infectious trigger or pre-existing tumor-suppressor gene mutations in an ILC3-dependent manner [112]. Moreover, genes commonly associated with polymorphisms in IBD and colitis-associated cancer (CAC)—such as *Card9*—are required to control ILC3 activation and IL-22 production via regulation of the ILC3 activating cytokine IL-1β [113]. These findings suggest dysregulation of the ILC3 and/or Th17 axis likely plays critical roles in the onset and progression of colorectal cancer.

In line with this, ILC3-associated cytokines have been heavily implicated in the pathogenesis of CRC but have been attributed conflicting roles—indicating the complexity of ILC3 biology in health and disease. For example, both IL-17A and IL-22 have been associated with human CRC and accumulation of IL-17-producing cells has been shown to be an independent prognostic marker in human CRC [112, 114]. Importantly, polymorphisms in IL-22 are associated with an increased risk for development of CRC [115]. As covered earlier in this review, ILC3 are a dominant and potent source of IL-22, which acts to directly regulate IEC function and proliferation. In human CRC, both T cell and non-T cell (lineage-negative) sources of IL-22 have been observed [116, 117]; however, in a model of bacteria-driven colitis-associated cancer (CAC) colonic ILC3 were sufficient for the development of invasive tumors [116]. In this case, blockade of IL-17 led to a reduction in inflammation; however, neutralization of IL-22 led to abrogation of STAT3 phosphorylation in the epithelium and a reduction in dysplasia [116]. In line with this, mice lacking the IL-22 binding protein (IL-22BP)—which regulates IL-22 bioactivity—show increased tumorigenesis, further suggesting a pro-tumorigenic role for chronic overexpression of IL-22 [118]. Despite these findings, the role of ILC3 and IL-22 in cancer appears to be highly contextual. Indeed, ILC3 have been attributed differential roles in disease outcome in lung cancer [119], breast cancer [120], and skin cancer [121] and the tissue and tumor microenvironment has been demonstrated to be critical in determining how ILC3 effect tumor growth [122].

The contradictory roles of ILC3 in cancer are likely also explained by the stage of disease. For example, while early loss of IL-22 results in higher tumor loads in a colitis and chemical carcinogen-driven mouse model, as well as in mice with a genetic lesion in the APC tumor suppressor gene (APC^min^ mice) [118], neutralization of IL-22 after the peak of colitis-induced tissue damage resulted in reduced growth and proliferation of tumors [108, 118]. These seemingly contradictory observations can be explained by the biology of IL-22R signaling on IECs, which acts to regulate cellular proliferation but is also required for homeostatic epithelial cell renewal and maintenance of the stem cell niche [38–40, 123]. Thus, regulation of intestinal tolerance by ILC3 is critical not only to prevent inflammation but also to prevent the development and growth of colorectal tumors (Fig. 2). Further understanding of how and when ILC3 regulate tumorigenesis may provide novel intervention strategies for CRC patients in the future.

### Graft-versus-host disease

Maintenance and restoration of gastrointestinal homeostasis is also central in the management of pathologies arising as a complication of clinical intervention. Allogeneic hematopoietic cell transplantation (HCT) is a potentially curative treatment strategy for hematological malignancies [124]. However, donor cells can become activated against recipient antigens, leading to immune-mediated tissue damage in organs including the skin, liver, and GI tract—a clinical entity known as graft-versus-host disease (GVHD). Loss of intestinal immune homeostasis is central to GVHD, as conditioning chemotherapy and/or radiotherapy given prior to allogeneic HCT to debulk the malignancy and to prevent rejection also leads to disruption of intestinal barrier integrity [124, 125]. Subsequently, translocation of gut commensals can lead to activation of donor T cells and acute GVHD (aGVHD), which occurs within 3 months of HCT [124, 125]. In acute myeloid leukemia, patients with higher circulating numbers of activated ILC3 following conditioning chemotherapy were found to have a lower incidence of aGVHD [126]. This suggests that ILC3s may protect against tissue damage following conditioning chemotherapy and impede the development of aGVHD. This is supported by a role for ILC3-derived IL-22 in mouse models of GVHD. Transplant-recipient mice lacking IL-22 exhibit increased intestinal epithelial cell damage, disruption of epithelial barrier integrity, and loss of intestinal crypt stem cells [38]. ILC3-derived IL-22 acts to protect stem cells from chemical damage-induced apoptosis and promotes regeneration of the stem cell niche following GVHD-induced depletion [38–40]. Thus, the ILC3–IL-22 axis can act as a critical tolerogenic factor in the intestine to prevent transplant-associated damage and disease. However, as in IBD and CRC, the effects of ILC3 and IL-22 on the outcome of GVHD are likely to be contextual and contrasting studies have demonstrated a role for donor-derived IL-22 in exacerbating inflammation and tissue damage in GVHD [127]. Thus, while targeting of ILC3-associated pathways may yet prove efficacious in treatment of GVHD, further work is needed to better understand the optimal window for intervention and to clarify apparently contrasting effects of this axis in patients.

### HIV and infection-driven loss of intestinal tissue homeostasis

While loss of intestinal homeostasis can be driven by genetic lesions, environmental factors, or treatment-associated side effects that precipitate inflammatory disease, a perhaps more common trigger for dysregulated intestinal homeostasis is infection. Indeed, disruption of ILC3 function is increasingly being demonstrated following infectious insult. Viral infections in particular are thought to disrupt tolerance and drive sensitization to dietary and microbial antigens in the intestine [128]. Moreover, a dramatic loss of ILC3 and intestinal barrier integrity is observed in immunodeficiency virus infections of primates and humans (e.g., SIV, HIV) [129–133]. HIV-1 infection in humans in particular is associated with impaired gut barrier and translocation of commensal bacteria which drive systemic inflammation [134]. While profound depletion of infected CD4^+^ T cells is the hallmark of HIV-1 infection, recent studies have demonstrated dysregulation of ILC3 in the blood, lymph, and/or intestinal tissues of HIV-1-infected humans or humanized mice and SIV-infected macaques [130–133]. Thus, in the setting of HIV or SIV infection, loss of tissue-protective ILC3 responses may lead to loss of mucosal tolerance towards gut commensals, propagating intestinal and systemic inflammation. In support of this, loss of circulating ILC frequencies coincides with the elevation of markers associated with gut barrier breakdown and bacterial translocation [130, 135]. In addition, SIV-infected macaques have reduced frequencies of small intestinal IL-17-producing ILCs following initial and persisting infection [132]. Notably, ILC3 depletion in HIV/SIV infection does not appear to be due to direct viral infection [130, 136]. Rather, mechanistic studies have suggested that ILC3 depletion is the result of apoptosis, possibly due to dysregulation of ILC survival signals [137]. As such, analysis of ILC3 from humans with HIV-1 viremia shows upregulation of genes linked to apoptosis and cell death, including CD95 (Fas) [130]. In humanized mice, CD95 expression on ILC3s can be induced by plasmacytoid DC-derived IFNα, which is also upregulated in the setting of HIV-1 infection [133]. Thus, infection-induced cues likely impact upon ILC3 survival. Alternatively, loss or dysregulation of ILC3 responses may rather be a consequence of the depletion of CD4^+^ T cells associated with HIV/SIV infection. As detailed above, ILC3 engage in multiple interactions with CD4^+^ T cells and while the effect on T cells has been investigated in some detail, the consequences of these interactions for ILC3 remain unclear. In line with this possibility, absence of T cells has been associated with dysregulation of ILC3 function in the intestinal tissue [138, 139], while *Tcra*^−/−^ mice exhibit a reduction in ILC3 numbers in the gut-draining mLN [21]. Thus, it is tempting to speculate that HIV-induced depletion of T cells in infected patients may precipitate loss of ILC3s. Further work in this area may help delineate the nature of ILC3 and CD4^+^ T cell interactions and increase understanding of how infection can result in a dramatic loss of ILC3 responses and precipitate inflammation in the intestine.

Moreover, it is likely viral infection may result in a loss of tolerance not only to commensal bacterial antigens but also to other environmental and dietary antigens (Fig. 2). For example, infection with reovirus has recently been demonstrated to drive sensitization to gluten and the development of celiac disease [128]. Similarly, dysregulated ILC3 function and IL-22 secretion have been reported in celiac disease [65, 140]; however, further work is needed to determine whether disruption of ILC3 responses is a common pathway in sensitization and inflammation against a wide range of microbial, environment, and dietary factors.

## Conclusions

The maintenance of tolerance and immune homeostasis in the gastrointestinal tract is critical for mammalian health. This process is mediated by a broad range of non-immune and immune cell types, and in this review, we have highlighted the various functions of group 3 innate lymphoid cell (ILC3) subsets in regulating gastrointestinal health. Disruption of beneficial ILC3 functions or dysregulation of ILC3 phenotype resulting from genetic polymorphisms and environmental insults is central to a broad range of clinically relevant pathologies. As such, there is an increasing interest in developing novel therapeutics aimed at restoring or enhancing tolerogenic and homeostatic ILC3 functions while suppressing their pro-inflammatory effector pathways. The emerging pipeline of monoclonal antibody therapies and small-molecule agonists and antagonists that target the related Th17 pathway may allow rapid integration of ILC3-targeting therapeutics into the clinic and increase awareness of the importance of these cells not only in the treatment of chronic inflammatory disease but also in predicting the long-term consequences of immunotherapeutics.
